# *Ancistrocladus tectorius* Extract Inhibits Obesity by Promoting Thermogenesis and Mitochondrial Dynamics in High-Fat Diet-Fed Mice

**DOI:** 10.3390/ijms25073743

**Published:** 2024-03-27

**Authors:** Minju Kim, Jin Hyub Paik, Hwa Lee, Min Ji Kim, Sang Mi Eum, Soo Yong Kim, Sangho Choi, Ho-Yong Park, Hye Gwang Jeong, Tae-Sook Jeong

**Affiliations:** 1 Microbiome Convergence Research Center, Korea Research Institute of Bioscience and Biotechnology (KRIBB), Daejeon 34141, Republic of Korea; qozus0@kribb.re.kr (M.K.); leehua@kribb.re.kr (H.L.); kam4256@naver.com (M.J.K.); hypark@kribb.re.kr (H.-Y.P.); 2 College of Pharmacy, Chungnam National University, Daejeon 34134, Republic of Korea; 3 International Biological Material Research Center, Korea Research Institute of Bioscience and Biotechnology (KRIBB), Daejeon 34141, Republic of Korea; jpaik@kribb.re.kr (J.H.P.); eomsm@kribb.re.kr (S.M.E.); soodole@kribb.re.kr (S.Y.K.); decoy0@kribb.re.kr (S.C.)

**Keywords:** *Ancistrocladus tectoirus*, anti-obesity, brown adipose tissue, thermogenesis, mitochondrial dynamics

## Abstract

Root extracts of *Ancistrocladus tectorius* (AT), a shrub native to China, have been shown to have antiviral and antitumor activities, but the anti-obesity effects of AT aerial parts, mainly the leaves and stems, have not been investigated. This study is the first to investigate the anti-obesity effects and molecular mechanism of AT 70% ethanol extract in 3T3-L1 adipocytes and high-fat diet (HFD)-fed C57BL/6J mice. Treatment with AT extract inhibited lipid accumulation in 3T3-L1 cells and decreased the expression of adipogenesis-related genes. AT extract also upregulated the mRNA expression of genes related to mitochondrial dynamics in 3T3-L1 adipocytes. AT administration for 12 weeks reduced body weight and organ weights, including liver, pancreas, and white and brown adipose tissue, and improved plasma profiles such as glucose, insulin, homeostasis model assessment of insulin resistance, triglyceride (TG), and total cholesterol in HFD-fed mice. AT extract reduced HFD-induced hepatic steatosis with levels of liver TG and lipogenesis-related genes. AT extract upregulated thermogenesis-related genes such as *Cidea*, *Pgc1α*, *Ucp1*, *Prdm16*, *Adrb1*, and *Adrb3* and mitochondrial dynamics-related genes such as *Mff*, *Opa1*, and *Mfn2* in brown adipose tissue (BAT). Therefore, AT extract effectively reduced obesity by promoting thermogenesis and the mitochondrial dynamics of BAT in HFD-fed mice.

## 1. Introduction

Obesity is characterized by an increase in the number of adipocytes (hyperplasia) and an increase in adipocyte size (hypertrophy) due to the accumulation of triglycerides (TG) [1,2]. Increased TG storage in adipocytes can lead to several metabolic diseases, such as diabetes and cardiovascular disease [3]. The fundamental cause of obesity is an imbalance between excess energy storage and insufficient energy expenditure [4]. There are three types of adipose tissue (AT) in humans: white adipose tissue (WAT), beige adipose tissue, and brown adipose tissue (BAT). The main function of WAT, the most important organ in the body, is to store excess energy in the form of TG [5]. In contrast, BAT causes energy expenditure through thermogenesis mediated by uncoupling protein 1 (UCP1) [6]. UCP1 activates the inner mitochondrial membrane of BAT to uncouple mitochondrial respiration from ATP biosynthesis and generate heat [7]. BAT improves glucose clearance and energy expenditure and inhibits fibrosis [8]. Thermogenesis in BAT is being investigated as a primary target and strategy for the treatment of obesity. Brown adipocytes have multiple small lipid droplets and contain numerous mitochondria [6]. Mitochondria maintain metabolic homeostasis through the processes of fusion and fission, known as mitochondrial dynamics [9]. Mitochondrial dynamics are regulated by related dynamics factors. Mitochondrial fusion is regulated by dynamin-related fusion factors such as mitofusin 1 (MFN1), mitofusin 2 (MFN2), and the dynamin-related protein optic atrophy protein 1 (OPA1) [10,11]. Mitochondrial fission is controlled by the fission-related genes dynamin 1 (DNM1), dynamin 2 (DNM2), and mitochondrial fission factor (MFF) [11]. In obesity, dysfunction of mitochondrial dynamics may exacerbate dysfunction and affect cellular metabolism.

*Ancistrocladus tectorius* (Lour.) Merr. (AT) is found in Cambodia, China, India, Indonesia, Laos, Malaysia, Myanmar, Singapore, Thailand, and Vietnam, and belongs to the family *Ancistrocladaceae*. AT root extract is used in the traditional treatment of dysentery, malaria, and parasitic infections in China and Malaysia [12,13,14]. In addition, AT root extract has been shown to have antiviral and antitumor activity [15,16] and to improve blood circulation [13]. Naphthylisoquinoline alkaloids with antiplasmodial activity have been isolated from AT stems and twigs [17,18]. In addition, other biological effects of AT leaf, bark, and root extracts have been investigated, including antibacterial [19], antifungal [19], antiparasitic [13], and antispasmodic [12] activities. Since the 70% EtOH extract of AT has the inhibitory effect of suppressing lipid accumulation in 3T3-L1 adipocytes, we hypothesized that aerial parts of AT (mainly the leaves and stems) may be useful in the prevention or treatment of obesity. In this study, the anti-obesity effect of AT extract and its molecular mechanisms were investigated in high-fat diet (HFD)-fed C57BL/6J mice. To elucidate the molecular mechanism underlying the anti-obesity effect of AT extract in diet-induced obese mice, we evaluated the reduction of body and fat weight, plasma profiles, glucose homeostasis, and adipocyte hypertrophy in WAT, BAT, and liver. The expression of genes related to thermogenesis and mitochondrial dynamics were also examined.

## 2. Results

### 2.1. HPLC Analyses of AT Extract and its Butanol (BuOH) Fraction

The high-performance liquid chromatography (HPLC) profiles of the 70% EtOH extract of AT and its BuOH fraction were detected using a diode array detector (DAD) at 335 nm (Figure S1). The retention times and UV spectra of compound **1** in the AT extract and its BuOH fraction were the same as those in the HPLC profiles. As a result of analysis by HPLC-MS/MS (Table S1 and Figures S2 and S3), compound **1** was predicted to be ancistrocladinium A, a naphthylisoquinoline alkaloid produced by *Ancistrocladus tectorius* [https://pubchem.ncbi.nlm.nih.gov/compound/15984091, ancistrocladinium A (accessed on 23 July 2023); [12,16,20]. The peak areas of compound **1** in AT 70% ethanol extract and its BuOH fraction were 1,868,790 ± 28,096 and 11,083,858 ± 184,849, respectively. The peak area of compound **1** in the BuOH fraction was 5.9 times higher than that in the AT 70% ethanol extract.

### 2.2. Effect of AT Extract and Its BuOH Fraction on Cytotoxicity and Adipogenesis in 3T3-L1 Cells

**Figure 1:**
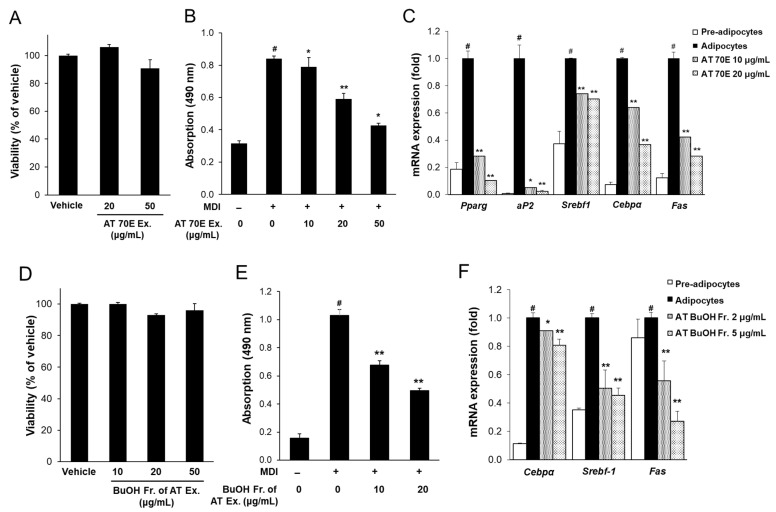
Effect of AT extract and its BuOH fraction on cytotoxicity and adipogenesis in 3T3-L1 adipocytes. Cytotoxicity of AT extract (**A**) and its BuOH fraction (**D**) in 3T3-L1 cells. (**B**) Quantification of lipid accumulation of AT extract (**B**) and its BuOH fraction (**E**) in differentiated adipocytes assessed by Oil Red O staining. The stained lipid was extracted from the cells with isopropanol and its absorbance was monitored spectrophotometrically at 490 nm. (**C**,**F**) Relative mRNA levels of adipogenesis-related genes were determined 4 days after sample treatment. AT 70E: 70% EtOH extract of AT; AT BuOH Fr.: BuOH fraction of AT 70% EtOH extract. Values are presented as means ± SD. ^#^ *p* < 0.01 versus pre-adipocytes, * *p* < 0.05, ** *p* < 0.01 versus adipocytes.

The effects of AT extract on lipid accumulation were evaluated in 3T3-L1 adipocytes. AT extract did not exert any cytotoxicity in 3T3-L1 adipocytes at 10 and 20 μg/mL (Figure 1A). Oil red O staining showed that the treatment with AT extract during adipocyte differentiation inhibited lipid accumulation at 10 and 20 μg/mL (Figure 1B). The effect of AT extract on the expression of adipogenesis-related genes was also evaluated in 3T3-L1 cells. Treatment with 10 and 20 μg/mL AT extract decreased the expression of the adipogenesis-related genes, including peroxisome proliferator-activated receptor gamma (*Pparg*), CCAAT/enhancer-binding protein (*Cebpα*), fatty acid binding protein 4 (*Fabp4*, known as aP2), sterol-regulated element-binding protein 1 (*Srebf1*), and fatty acid synthase (*Fas*) (Figure 1C). These results suggest that AT extract treatment reduces lipid accumulation by down-regulating the expression of adipogenesis-related genes.

In this study, the effects of the BuOH fraction of AT extract with high ancistrocladinium A content on lipid accumulation in 3T3-L1 adipocytes were investigated. The BuOH fraction showed no cytotoxicity on 3T3-L1 cells up to 50 μg/mL (Figure 1D). Compared to AT extract, treatment with BuOH fraction at 10 and 20 μg/mL strongly inhibited lipid accumulation in 3T3-L1 adipocytes (Figure 1E).

The effect of the BuOH fraction on the expression of adipogenesis-related genes was also investigated. When the BuOH fraction was treated with 10 and 20 μg/mL, almost no expression of adipogenesis-related genes was observed. Treatment with the BuOH fraction at relatively low concentrations of 2 and 5 μg/mL also significantly reduced the expression of *Cebpα*, *Srebf-1*, and *Fas* genes (Figure 1F).

### 2.3. Effect of AT Extract and Its BuOH fraction on Mitochondrial Dynamics in Mature 3T3-L1 Adipocytes

**Figure 2:**
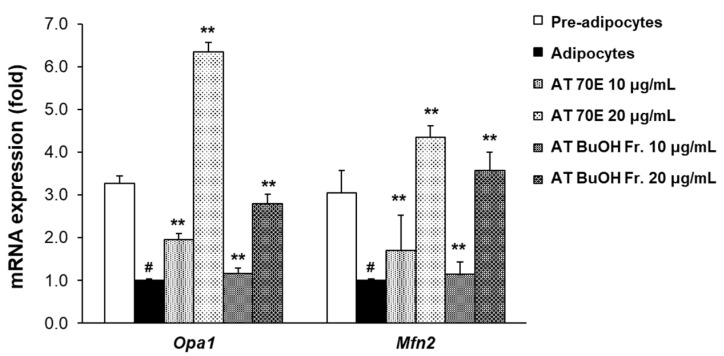
Effect of the AT extract and its BuOH fraction on genes related to mitochondrial dynamics in mature 3T3-L1 adipocytes. Two days after confluence, 3T3-L1 pre-adipocytes (day 0) were treated with samples at 10 or 20 μg/mL every other day for 4 days. The mRNA expression was measured on fully differentiated adipocytes (Day 8). AT 70E: 70% EtOH extract of AT; AT BuOH Fr.: BuOH fraction of AT 70% EtOH extract. Values are expressed as mean ± SD. ^#^ *p* < 0.01 versus pre-adipocytes, ** *p* < 0.01 versus adipocytes.

In BAT, energy expenditure during thermogenesis occurs via mitochondria. Upregulation of thermogenic activity increases the demand for mitochondria. Mitochondria are maintained by fission and fusion depending on the metabolic environment of the cells, and this is referred to as mitochondrial dynamics [4]. Mitochondrial dysfunction is associated with obesity, diabetes, and various metabolic diseases [21]. Therefore, this study measured the mRNA levels of mitochondrial dynamics-related fusion factors such as OPA1 and MFN2 in mature 3T3-L1 adipocytes. Treatment with AT extract and its BuOH fraction at 10 and 20 μg/mL increased the mRNA expression of mitochondrial dynamics-related genes, including *Opa1* and *Mfn2*, compared to mature adipocytes (Figure 2). These results showed that treatment with the AT BuOH fraction at the same concentration as mature 3T3-L1 adipocytes had slightly less activity than AT extract in terms of affecting mitochondrial dynamics related fusion factors.

### 2.4. Effect of AT 70% EtOH Extract on Body and Organ Weights

**Figure 3:**
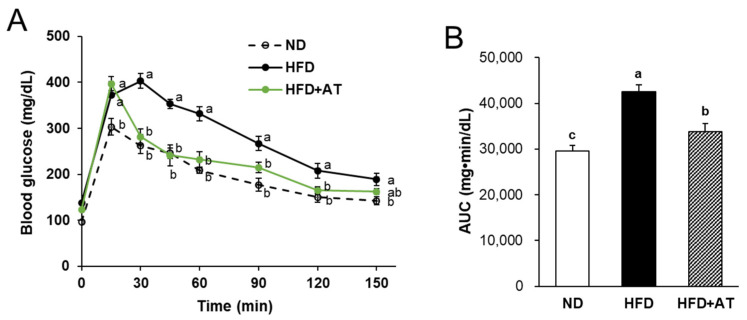
Effect of AT extract on oral glucose tolerance test (OGTT) in HFD-fed mice. (**A**) Blood glucose levels were measured in tail vein blood at 0, 15, 30, 45, 60, 90, 120, and 150 min. (**B**) Area under the curve (AUC) of changed glucose levels during OGTT. Data are expressed as mean ± SE (*n* = 10). Different letters (a, b, c) within a variable are significantly different at *p* < 0.05.

In the in vivo experiment, the C57BL/6 mice were fed an HFD diet for 12 weeks to induce obesity. Changes in body weight and food consumption were monitored weekly. The body weights among all the groups were not different at the initiation of this study. After 12 weeks of feeding, the body weight of the HFD group was significantly higher than that of the ND group (Figure S5). However, the weight of the HFD + AT group was significantly lower than that of the HFD group (by 13.6%) (Figure 3B). In the in vivo experiments, the administration of AT extract did not affect the food intake of HFD mice (Table 1).

After 12 weeks, the weights of the liver, pancreas, total WAT, and BAT in the HFD group were significantly higher than those in the ND group (Table 1). However, AT extract treatment resulted in a significant decrease in organ weight including liver (by 31.8%), pancreas (by 20.9%), retroperitoneal AT (by 12.2%), inguinal AT (by 15.2%), total WAT (by 8.2%), and BAT (by 17.6%) compared to the HFD group (Table 1). Therefore, these results showed that AT extract treatment reduced the body and organ weights of the HFD mice.

### 2.5. Effect of AT Extract on Glucose Homeostasis and Plasma Profiles

After 10 weeks of feeding, an oral glucose tolerance test (OGTT) was performed to determine the effect of AT on glucose homeostasis in HFD-fed mice. The OGTT data showed significant glucose intolerance in the HFD group compared to the ND group (Figure 3A). Blood glucose levels in the HFD + AT group were significantly lower than those in the HFD group at 30, 60, 90, 120, and 150 min after glucose administration. The area under the curve (AUC) for integrated glucose concentration was significantly reduced (by 20.4%) in the HFD + AT group compared to the HFD group (Figure 3B). The AUC results indicated that the administration of AT extract effectively improved glucose tolerance.

At the endpoint (12 weeks) of the experiment, the plasma profiles of glucose, insulin, homeostasis model assessment of insulin resistance (HOMA-IR), triglycerides (TG), total cholesterol (TC), aspartate aminotransferase (AST), and alanine aminotransferase (ALT) were significantly increased in the HFD group (Table 2). However, AT extract supplementation decreased the glucose, insulin, and HOMA-IR levels by 21.5%, 15.6%, and 35.8%, respectively, in HFD-fed mice. In addition, AT extract administration reduced TG and TC concentrations (by 38.6% and 22.1%, respectively) compared to those in the HFD group. AST and ALT, liver function parameters, were significantly lower in the HFD + AT group than in the HFD group (by 26.1% and 56.2%, respectively). Administration of AT extract improves plasma metabolic parameters, and these results suggest that AT extract may have moderating effects on hyperglycemia and hyperlipidemia.

### 2.6. Effect of AT Extract on Regulation of Hepatic Steatosis

**Figure 4:**
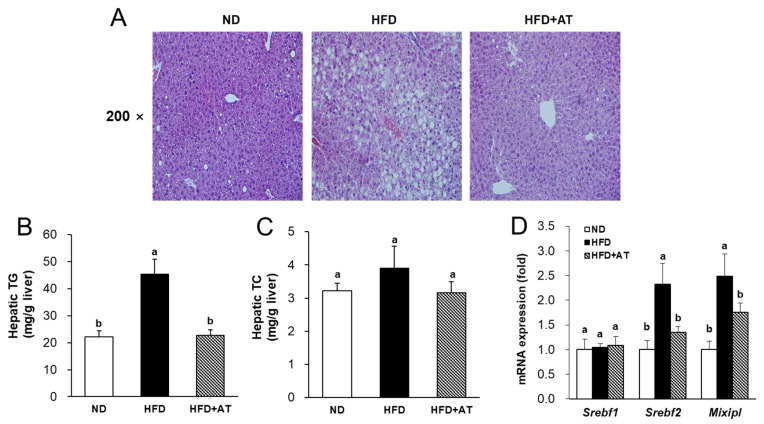
Effect of AT extract on hepatic lipid metabolism in HFD-fed mice. (**A**) Histology of livers stained with hematoxylin and eosin (H&E) (×200 magnification); (**B**) hepatic TG and (**C**) TC levels; (**D**) Lipogenesis-related mRNA expression levels were measured by real-time qRT-PCR. Data are expressed as mean ± SE. Different letters (a, b) within a variable are significantly different at *p* < 0.05.

The effects of AT extract on hepatic lipid metabolism were then assessed. Livers were fixed in a 10% formalin and embedded in paraffin blocks. The paraffin blocks were cut sectioned and stained with hematoxylin and eosin (H&E). The number and size of hepatic lipid droplets in the HFD group were significantly higher than in the ND group, indicating hepatic steatosis (Figure 4A). However, AT supplementation reduced the number and size of hepatic lipid droplets compared to those in the HFD group. In addition, hepatic TG content was significantly reduced (by 50%) in the HFD + AT group compared to the HFD group (Figure 4B). In contrast, AT administration had no effect on hepatic TC content (Figure 4C).

Next, transcription factors and target genes related to lipogenesis in the liver were examined. As shown in Figure 4D, AT extract treatment downregulated the mRNA levels of element-binding transcription factor 2 (*Srebf2*) and MLX-interacting protein-like (*Mlxipl*) in the liver of the HFD + AT group compared to the HFD group (Figure 4D). However, AT administration had no effect on *Srebf 1* levels (Figure 4D). These results suggest that AT extract prevents HFD-induced hepatic steatosis.

### 2.7. Effect of AT Extract on Adipocyte Hypertrophy in Adipose Tissues

**Figure 5:**
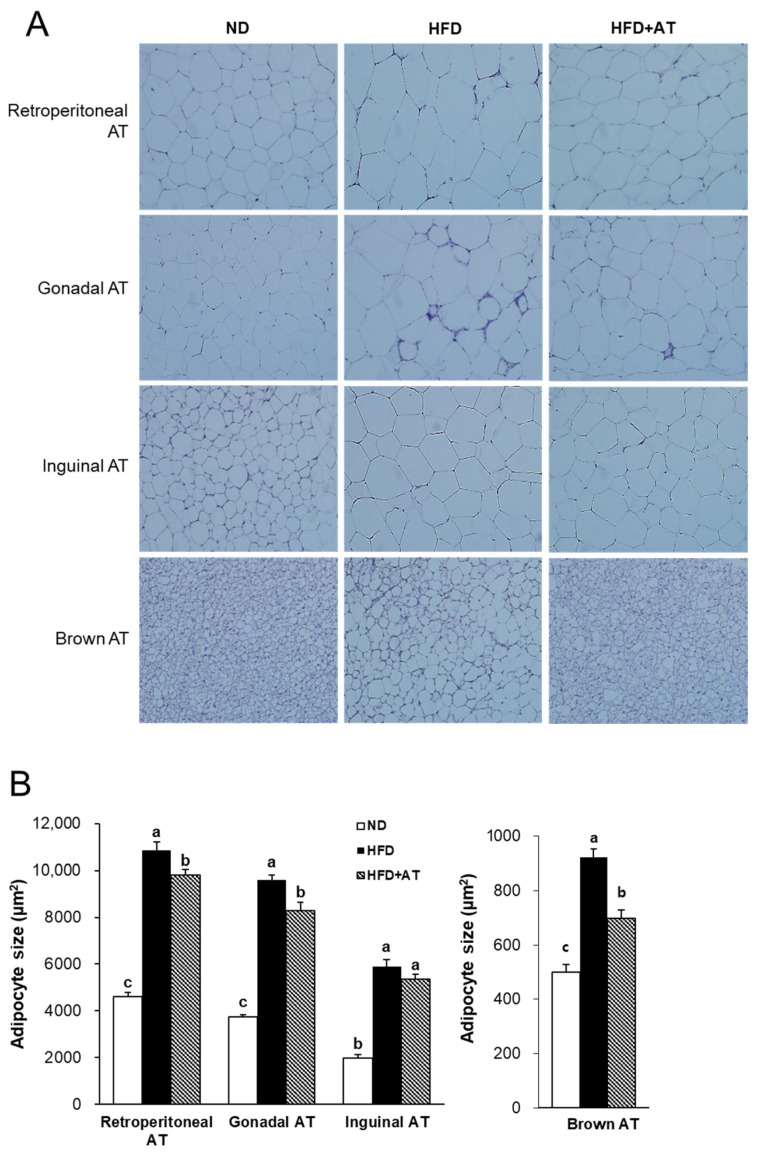
Effects of AT extract on adipocyte hypertrophy in HFD-fed mice. (**A**) Histology of WAT and BAT stained with H&E (200× magnification). (**B**) Quantitative measurement of adipocyte size. Data are expressed as mean ± SE. Different letters (a, b, c) within a variable are significantly different at *p* < 0.05.

H&E staining was performed to analyze the morphology and adipocyte size of the inguinal, retroperitoneal, gonadal, and brown adipocytes. The inguinal, retroperitoneal, and gonadal adipocyte sizes were significantly increased in the HFD group compared to the ND group (Figure 5A). However, AT extract supplementation significantly decreased the adipocyte size in retroperitoneal and gonadal WAT, but not in inguinal WAT (Figure 5A,B). In addition, AT extract treatment significantly reduced the size of brown adipocytes (Figure 5A,B). These results showed that AT extract inhibited adipocyte hypertrophy in HFD-fed mice.

### 2.8. Effect of AT Extract on Thermogenesis of BAT in HFD-Induced Obese Mice

**Figure 6:**
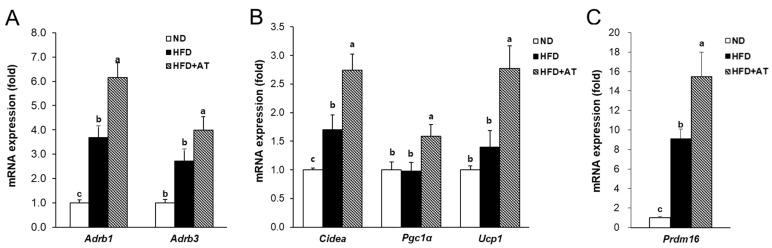
Effect of AT extract on the thermogenesis in BAT of HFD-fed mice. (**A**–**C**) The mRNA expression levels were measured by real-time qRT-PCR in the BAT. Data are expressed as mean ± SE. Different letters (a, b, c) within a variable are significantly different at *p* < 0.05.

BAT causes energy expenditure through thermogenesis and is the primary target as a strategy for obesity treatment [22]. To investigate the anti-obesity effect of AT extract, the thermogenesis mechanism was investigated using the key thermogenesis-related markers of BAT. Administration of AT extract reduced brown adipocyte size and weight compared with the HFD-induced group (Figure 5 and Table 1). These results suggest the possibility that AT extract stimulates the expression of adrenergic receptors. AT extract treatment increased the mRNA levels of adrenergic receptors, including beta-1 adrenergic receptor (*Adrb1*) and beta-3 adrenergic receptor (*Adrb3*), in BAT of the HFD + AT group (Figure 6A). Furthermore, the mRNA expression of thermogenesis-related genes, such as uncoupling protein 1 (*Ucp1*), peroxisome proliferator-activated receptor-gamma coactivator (*Pgc1α*), PR domain containing 16 (*Prdm16*), and cell death-inducing DFFA-like effector a (*Cidea*), were observed in BAT. Treatment with AT extract promoted thermogenesis by upregulating the mRNA levels of *Cidea*, *Pgc1α*, *Ucp1*, and *Prdm16* (Figure 6B,C).

### 2.9. Effect of AT Extract on BAT Mitochondrial Dynamics in HFD-Fed Mice

**Figure 7:**
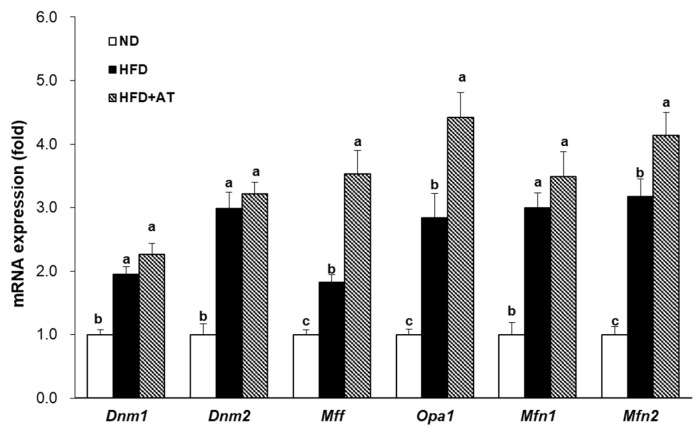
Effect of AT extract on the mitochondrial dynamics in BAT of HFD-fed mice. The mRNA expression levels were measured by real-time qRT-PCR in the BAT. Data are expressed as mean ± SE. Different letters (a, b, c) within a variable are significantly different at *p* < 0.05.

Treatment with AT extract significantly increased the expression of the mitochondrial dynamics related factors *Opa1* and *Mfn2* in 3T3-L1 cells (Figure 2). Therefore, it was investigated whether AT extract contributes to the expression of mitochondrial dynamics-related genes in the BAT of HFD-fed mice. Supplementation with AT extract improved mitochondrial dynamics by upregulating the mRNA expression of *Mff*, *Opa1*, and *Mfn2* in BAT compared to HFD-induced mice (Figure 7). These results suggest that the AT extract enhances mitochondrial dynamics in the BAT of HFD-fed mice. However, AT administration had no effect on the levels of *Dnm1*, *Dnm2*, or *Mfn1* (Figure 7).

## 3. Discussion

AT extract has been traditionally used to treat malaria and dysentery and to improve blood circulation [12,13,14]. In addition to these functions, some studies have reported biological activities such as antiplasmodial [17,18], antiparasitic [13], antiviral [15], and antibacterial [18] activities for AT. In this study, the anti-obesity effect of 70% EtOH extract from the aerial parts of AT, mainly the leaves and stems, and its molecular mechanisms were investigated in 3T3-L1 adipocytes and HFD-fed C57BL/6J mice.

Treatment with AT extract at 20 μg/mL inhibited lipid accumulation in 3T3-L1 cells (Figure 1B). In addition, treatment with AT extract significantly decreased the levels of adipogenesis-related genes, including *Pparg*, *aP2*, *Srebf1*, *Cebpα*, and *Fas*, in 3T3-L1 adipocytes (Figure 1C). In addition, treatment of the BuOH fraction of AT extract at 2 and 5 μg/mL significantly inhibited lipid accumulation (Figure 1E) and significantly decreased the gene levels of *Cebpα*, *Srebf1*, and *Fas* in 3T3-L1 adipocytes (Figure 1F). These results showed that the AT BuOH fraction with high ancistrocladinium A content was more effective in suppressing the expression of adipogenesis-related genes than the 70% EtOH extract of AT.

Treatments with AT extract and its BuOH fraction at the same concentrations (10 and 20 μg/mL) increased the mRNA expression of Opa1 and Mfn2 in mature 3T3-L1 adipocytes (Figure 2). In contrast to the effect of the low concentration BuOH fraction, which strongly suppressed the expression of adipogenesis-related genes, the gene levels of mitochondrial dynamics-related fusion factors in the AT BuOH fraction were slightly lower than those in the AT extract at the same concentration. Therefore, our results indicate that high levels of ancistrocladinium A in the BuOH fraction are not effective against mitochondrial dynamics-related fusion factors in mature 3T3-L1 adipocytes.

Subsequent in vivo experiments evaluated the anti-obesity and anti-diabetic effects of AT extract in HFD-induced C57BL/6J mice for 12 weeks. In general, obesity is a result of overnutrition, which leads to weight gain due to the presence of excess body fat. Using a high-fat diet (HFD)-fed obese mouse model, we confirmed the HFD-induced obese condition compared with the ND group by examining the parameters of obesity and hyperglycemia, such as increased body weight, fat weight, and lipid profiles. However, when we investigated the molecular mechanisms underlying the anti-obesity effects of AT extract, we did not use the ND group in order to compare the same HFD-fed conditions (HFD and HFD + AT groups). In animals treated with HFD + AT for 12 weeks, there were no toxic effects visible to the naked eye, and no abnormalities were found in organs dissected at the end of the experiment. AT extract treatment reduced the body weight and WAT and BAT weights in the HFD-fed mice (Figure 5 and Table 1). Plasma profiles and OGTT showed glucose tolerance and hyperglycemia induced by HFD feeding. Administration of AT extract significantly reduced the plasma levels of glucose, insulin, HOMA-IR, TG, and TC (Figure S3 and Table 1). Administration of AT extract ameliorated HFD-induced hepatic steatosis induced with decreased expression of lipogenesis-related genes, including *Srebf2* and *Mlxipl* in the liver (Figure 4D). Therefore, this study found that AT extract exhibited anti-obesity effects by regulating hyperglycemia and lipogenesis in HFD-fed mice. In addition, AT extract significantly reduced AST and ALT in the HFD + AT group compared with the HFD group. This indicates that AT extract is effective in ameliorating hepatotoxicity caused by high-fat diet.

An imbalance between excessive food intake and energy expenditure causes obesity [23]. It is associated with a potential risk of type 2 diabetes, hypertension, and cardiovascular disease [24]. In obesity, WAT is adapted to store excess energy and expands by increasing adipocyte size (hypertrophy), which is associated with insulin resistance, decreased BAT content and activity, and mitochondrial dysfunction [25,26]. AT extract treatment reduced the weight and size of adipose tissue, including retroperitoneal WAT and gonadal WAT (Figure 5). In addition, AT extract treatment reduced the weight and size of brown adipocytes in BAT (Figure 5). Brown adipocytes have smaller lipid droplets than white adipocytes and contain numerous mitochondria, which cause energy expenditure and heat through increased expression of *UCP1* [27,28]. UCP1 is a protein in the inner membrane of mitochondria that uncouples oxidative phosphorylation from ATP production and releases the energy as heat [28]. Thermogenesis by activation of brown adipocytes is occurs through mechanisms such as β-adrenergic receptor stimulation in rodent BAT [29,30]. The activation of brown adipocytes increases BAT glucose and free fatty acid (FFA) uptake through *β*_3_-adrenergic receptor stimulation, thereby improving obesity or glucose metabolism [31]. In addition, a study found that inhibition of the ADRB3 adrenergic receptor in the BAT impaired thermogenesis and decreased the expression of *UCP1* [30].

This study found that AT extract treatment enhanced thermogenesis by stimulating the expression of *β*-adrenergic receptors. AT extract administration upregulated the expression levels of the adrenergic receptors, including *Adrb1* and *Adrb3*, and increased the levels of thermogenesis-related genes *Cidea*, *Ucp1*, *Pgc1α*, and *Prdm16* in the BAT of HFD-fed mice. The genes *Pgc1α* and *Prdm16* are associated with BAT thermogenesis, and their genetic loss can affect BAT formation and function [32]. *Cidea* is another gene that is expressed in the BAT and induces thermogenesis [33]. Accordingly, the current study demonstrated that AT extract can enhance thermogenesis by stimulating adrenergic receptors to promote the expression of BAT thermogenic genes, including *Cidea*, *Ucp1*, *Pgc1α*, and *Prdm16*.

Brown adipocytes burn lipids to produce heat and contain numerous mitochondria [34]. Mitochondria regulate metabolism, including heat production. Brown adipocytes have more mitochondria and higher mitochondrial dynamic activity than white adipocytes. Mitochondrial dynamics play an essential role in maintaining mitochondrial quality and function through fusion and fission [35]. Mitochondrial fusion occurs in the outer mitochondrial membrane by MFN1 and MFN2, and the inner mitochondrial membrane is bound by OPA1 to form long tubule mitochondria, which enhances ATP production and metabolic function [36,37]. The sequences of human Mfn1 and Mfn2 are about 80% similar but play different roles in mitochondrial dynamics. Outer mitochondrial membrane fusion is only completely inhibited when both mitofusin species are deleted [38]. In addition, Mfn1-KO induces mitochondrial fragmentation, whereas Mfn2-KO swells spherical mitochondria because Mfn1 mediates mitochondrial tethering more efficiently than Mfn2 [39,40]. In addition, mitochondrial fission is regulated by DNM2 and DRP1, which is associated with reduced oxidative phosphorylation, mtDNA depletion, and ROS production [21]. DNM1 (DRP1, dynamin-related protein 1) is activated by the MFF receptors on the outer mitochondrial membrane, leading to mitochondrial fission, which is facilitated by DNM2, also present on the outer membrane [11]. However, in a state of obesity, dysfunction in mitochondrial dynamics occurs, including reduced oxidative capacity and increased oxidative stress [36]. Maintaining homeostasis in mitochondrial dynamics is essential for normal organ function and its regulation is a target for obesity treatment.

Therefore, we investigated whether the administration of AT extract affects the regulation of mitochondrial dynamics. Supplementation with AT extract improved mitochondrial dynamics in BAT. Administration of AT extract upregulated the mRNA expression levels of the mitochondrial fusion genes *Mfn2* and *Opa1*, as well as the fission genes *Mff* in BAT. Furthermore, it has been reported that knockdown of *Mfn2* and *Opa1*, which are involved in mitochondrial dynamics, increased the accumulation of intracellular TG in 3T3-L1 cells [21,41]. Similarly, in our results, 3T3-L1 cells were treated with AT extract to examine the genes related to mitochondrial dynamics, which revealed increased expression of the genes involved in mitochondrial fusion, *Mfn2* and *Opa1.* These results suggest that AT extract has the effect of promoting the mitochondrial dynamics of BAT in HFD-fed mice.

**Figure 8:**
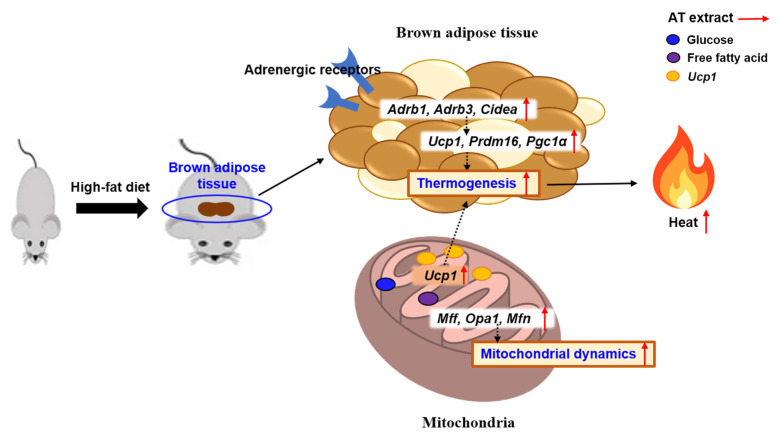
Molecular mechanisms of the anti-obesity effects of AT extract. AT extract suppresses obesity by enhancing thermogenesis and mitochondrial dynamics in BAT.

In summary, this study evaluated the effects of AT extract in HFD-fed obese and diabetic mice. The administration of AT extract reduced HFD-induced body weight and organ weights, including liver, pancreas, and white and brown adipose tissue, and reduced white and brown adipocyte size. AT extract administration improved the plasma profile of obesity and diabetes marker levels in mice. In addition, AT extract supplementation protected against hepatic steatosis by regulating the expression of lipogenesis-related genes and inhibited adipocyte hypertrophy in HFD-fed mice. The administration of AT extract stimulated the expression of adrenergic receptors in BAT, thereby enhancing thermogenesis by regulating the expression of thermogenesis-related genes. In addition, AT extract improved mitochondrial dynamics by increasing the levels of mitochondrial dynamics-related genes in BAT (Figure 8). Our results suggest that AT extract has an anti-obesity effect by regulating thermogenesis and mitochondrial dynamics, and that AT has the potential to be a novel nutraceutical for the treatment of diet-induced obesity.

## 4. Materials and Methods

### 4.1. Preparation of AT Extract and Its BuOH fraction

Aerial AT, mainly the leaf and stem, was harvested in Vietnam and supplied by the International Biological Material Research Center of the Korea Research Institute of Bioscience and Biotechnology (KRIBB, Daejeon, Republic of Korea). The dried leaves and stems of AT (50 g) were extracted with 70% ethanol (500 mL) at room temperature for 48 h. The extract was filtered and evaporated at a temperature below 40 °C to obtain a 70% ethanol extract of AT (408 mg). The 70% EtOH extract of AT was suspended in water (40 mL) and then subsequently partitioned with *n*-hexane, chloroform, ethyl acetate, and *n*-BuOH.

### 4.2. HPLC Analyses of AT Extract and Its Butanol (BuOH) Fraction

The AT extract and its BuOH fraction were analyzed using an HPLC system equipped with a diode array detector system (Shimadzu Corporation, Tokyo, Japan) and a Cosmosil 5C18 column (4.6 mm × 150 mm). The injection volume was 10 μL (10 mg/mL) and the mobile phase consisted of 0.1% acetic acid in water (solvent A) and acetonitrile (solvent B). The linear gradient elution program was set to 5% B for 0–5 min, 5–20% B for 5–15 min, 20–30% B for 15–27 min, 30–80% B for 27–35 min, 80–100% B for 35–40 min, 100% B for 40–45 min, and 100–5% B for 45–50 min. The flow rate was 1.0 mL/min and the absorbance of the HPLC profile was 335 nm.

### 4.3. Culture and Differentiation of 3T3-L1 Cells

3T3-L1 cells were obtained from the American Type Culture Collection (Rockville, MD, USA). 3T3-L1 cells were maintained in Dulbecco’s modified Eagle’s medium (DMEM) supplemented with 10% fetal calf serum (FBS) at 37 °C and in 5% CO_2_. To measure the mRNA profiles of AT extract, 3T3-L1 cells were incubated with a differentiation medium containing 0.5 μM 3-isobuty-1-methylxanthine, 5 μg/mL insulin, and 1 μM dexamethasone. After 2 days, the medium was changed to DMEM/FBS medium with 5 μg/mL insulin for further differentiation (day 4). The 3T3-L1 cells were incubated with AT extract or its BuOH fraction during the differentiation period. To determine the mRNA levels of genes related to mitochondrial dynamics, the 3T3-L1 adipocytes were replaced with DMEM/FBS medium and the cells were differentiated for 4 days (day 8).

### 4.4. Animals and Diets

All protocols in this animal study were approved by the Animal Care and Use Committee of KRIBB (KRIBB-AEC-22178). Four-week-old male C57BL/6J mice were obtained from the Laboratory Animal Resource Center of KRIBB. Mice were maintained at the facility under a controlled temperature (22 ± 2 °C), humidity (50 ± 5%), and lighting (12-h light/dark cycle) with free access to food and water in a dedicated pathogen-free facility at KRIBB. After a 3-week adaptation period, the mice were randomly divided into three groups (*n* = 10 per group): the normal diet (ND) group, which received a 10 kcal% normal diet (3.82 kcal/g, 10% fat, 20% protein, 70% carbohydrate) (D12450B; Research Diet, Inc., New Brunswick, NJ, USA); the HFD group, which was fed a 60 kcal% diet (5.21 kcal/g, 60% fat, 20% protein, 20% carbohydrate) (D12492; Research Diet, Inc., New Brunswick, NJ, USA) without supplementation; and an HFD-AT group, fed an HFD with oral administration of 100 mg/kg AT extract once a day for 12 weeks. The AT extract was dissolved in sterile purified water containing 10% polyethylene glycol and 0.5% Tween-80.

### 4.5. Measurement of Body Weight and Plasma Profiles

Body weight was monitored weekly for 12 weeks. At the end of the experiment, the mice were fasted overnight and blood was collected in EDTA-coated tubes. Plasma profiles such as fasting glucose, insulin, TG, TC, AST, and ALT concentrations were analyzed using commercially available kits (Asan Pharmaceutical, Seoul, Republic of Korea). Insulin levels were measured using a mouse insulin ELISA kit (Alpco Diagnostics, Salem, NH, USA). The insulin homeostasis model assessment of insulin resistance (HOMA-IR) was calculated as [fasting insulin concentration (ng/mL)] × 24.8 × [fasting glucose concentration (mg/dL)]/405.

### 4.6. Oral Glucose-Tolerance Test (OGTT)

After 10 weeks of supplementation, an OGTT was performed after the administration of glucose (2 g/kg). Blood glucose concentrations were measured at 0, 30, 50, 90, 120, and 150 min after glucose administration.

### 4.7. Histological Analysis

WAT, BAT, and liver were fixed in a 10% formalin solution and embedded into paraffin blocks. Tissue from the paraffin block was then sectioned at 4 μm sections and stained with H&E. Images of the stained tissues were obtained using an Olympus BX61 microscope system equipped with an Olympus DP71 digital camera (Tokyo, Japan). Adipocytes size was measured in three groups, including ND, HFD, and HFD + AT groups, using MetaMorph image analysis software (3 January 2023, Molecular Devices, Sunnyvale, CA, USA).

### 4.8. Quantitative Real-Time RT-PCR (qRT-PCR)

For RNA extraction, the 3T3-L1 cells or liver, WAT, and BAT were homogenized with TRI reagent (Ambion, Foster City, CA, USA), and total RNA was additionally purified using an RNeasy mini kit. cDNA was synthesized from total RNA using the high-capacity cDNA reverse transcription kit (Thermo Fisher Scientific, Inc., Waltham, MA, USA). The RNA expression levels were quantified by qRT-PCR using cDNA and SYBR Green Master Mix (Roche, Mannheim, Germany) on a 7500 Real-Time PCR System (Applied Biosystems, Foster City, CA, USA) with specific primers (Table S2).

### 4.9. Statistical Analysis

The data are expressed as mean ± standard error (SE). The statistical differences between groups were analyzed by one-way analysis of variance (ANOVA) with Tukey’s test using JMP^®^ software (22 August 2023, SAS Institute Inc., Cary, NC, USA). A *p* value < 0.05 was considered statistically significant.

## Figures and Tables

**Table 1:** Effect of AT extract on body weight and organ weights in HFD-fed mice.

	ND	HFD	HFD + AT
Initial body weight (g)	22.9 ± 0.3	23.2 ± 0.2	22.7 ± 0.4
Final body weight (g)	31.1 ± 0.5 ^c^	45.9 ± 1.0 ^a^	42.1 ± 1.1 ^b^
Body-weight gain (g)	8.2 ± 0.5 ^c^	22.8 ± 0.8 ^a^	19.7 ± 1.4 ^b^
Food intake (g/day)	2.4 ± 0.0	2.4 ± 0.1	2.4 ± 0.0
Liver (g)	1.08 ± 0.07 ^c^	1.51 ± 0.07 ^a^	1.03 ± 0.04 ^b^
Pancreas (g)	0.15 ± 0.01 ^c^	0.24 ± 0.01 ^a^	0.19 ± 0.02 ^b^
White adipose tissue (WAT) (g)			
Retroperitoneal AT	0.57 ± 0.05 ^c^	1.47 ± 0.06 ^a^	1.29 ± 0.05 ^b^
Gonadal AT	1.14 ± 0.05 ^b^	2.65 ± 0.09 ^a^	2.70 ± 0.10 ^a^
Inguinal AT	1.11 ± 0.06 ^c^	3.10 ± 0.08 ^a^	2.63 ± 0.25 ^b^
Total WAT	2.82 ± 0.14 ^c^	7.21 ± 0.15 ^a^	6.62 ± 0.35 ^b^
Brown adipose tissue (g)	0.22 ± 0.02 ^b^	0.34 ± 0.03 ^a^	0.28 ± 0.04 ^b^

Data are indicated as means ± SE (*n* = 10). Different letters (a, b, c) indicate significant differences at *p* < 0.05.

**Table 2:** Effect of AT on plasma profiles in HFD-fed mice.

Parameters	ND	HFD	HFD + AT
Glucose (mg/dL)	93 ± 10 ^c^	163 ± 8 ^a^	128 ± 10 ^b^
Insulin (ng/mL)	1.1 ± 0.1 ^b^	3.2 ± 0.6 ^a^	2.7 ± 0.4 ^a^
HOMA-IR	5.4 ± 0.3 ^c^	31.6± 0.6 ^a^	20.3 ± 0.3 ^b^
TG (mg/dL)	93 ± 6 ^b^	161 ± 14 ^a^	99 ± 6 ^b^
TC (mg/dL)	179 ± 9 ^b^	235 ± 8 ^a^	183 ± 9 ^b^
AST (IU/L)	56 ± 13 ^b^	88 ± 10 ^a^	65 ± 6 ^b^
ALT (IU/L)	16 ± 8 ^b^	46 ± 7 ^a^	20 ± 2 ^b^

Data are indicated as means ± SE (*n* = 10). Different letters (a, b, c) indicate significant differences at *p* < 0.05.

## Data Availability

Data are included in the article and Supplementary Materials.

## Supplementary Materials

The following supporting information can be downloaded at: https://www.mdpi.com/article/10.3390/ijms25073743/s1.
