# Supplementary material for: Ancistrocladus tectorius Extract Inhibits Obesity by Promoting Thermogenesis and Mitochondrial Dynamics in High-Fat Diet-Fed Mice
Source: Int J Mol Sci. 2024 Mar 27;25(7):3743. doi: 10.3390/ijms25073743 (PMC11011498; doi:10.3390/ijms25073743)
Supplement: Supplementary file 1 [file ijms-25-03743-s001.zip › ijms-2914951-supplementary.pdf]

Supplementary file

Ancistrocladus tectorius Extract Inhibits Obesity by Promoting Thermogenesis and Mitochondrial Dynamics in High-Fat Diet-Fed Mice

Minju Kim [1,2], Jin Hyub Paik [3], Hwa Lee [1], Min Ji Kim [1], Sang Mi Eum [3], Soo Yong Kim [3], Sangho Choi [3], Ho-Yong Park [1], Hye Gwang Jeong [2,*] and Tae-Sook Jeong [1,*]

1 Microbiome Convergence Research Center, Korea Research Institute of Bioscience and Biotechnology (KRIBB), Daejeon 34141, Republic of Korea; qozus0@kribb.re.kr (M.K.); leehua@kribb.re.kr (H.L.); kam4256@naver.com (M.J.K.); hypark@kribb.re.kr (H.-Y.P.)
2 College of Pharmacy, Chungnam National University, Daejeon 34134, Republic of Korea
3 International Biological Material Research Center, KRIBB, Daejeon 34141, Republic of Korea; jpaik@kribb.re.kr (J.H.P); eomsm@kribb.re.kr (S.M.E.); soodole@kribb.re.kr (S.Y.K.); decoy0@kribb.re.kr (S.C.)
* Correspondence: hgjeong@cnu.ac.kr (H.G.J.); tsjeong@kribb.re.kr (T.-S.J.)

1. High-performance liquid chromatography (HPLC) analysis of AT 70% ethanol extract (AT extract) and its butanol (BuOH) fraction (Figure S1)

The AT extract and its BuOH fraction were analyzed using an HPLC system equipped with a diode-array detector system (Shimadzu Corporation, Tokyo, Japan) and a Cosmosil 5C18 column (4.6 mm × 150 mm). The injection volume was 10 μL (10 mg/mL), and the mobile phase was consisted of 0.1% acetic acid in water (solvent A) and acetonitrile (solvent B). The linear gradient elution program was set to 5% B for 0–5 min, 5–20% B for 5-15 min, 20–30% B for 15–27 min, 30–80% B for 27–35 min, and 80–100% B for 35–40 min, 100% B for 40–45 min, and 100–5% B for 45–50 min. The flow rate was 1.0 mL/min, and the absorbance of the HPLC profile was 335 nm.

As a result of analysis by HPLC-MS/MS (Tables S1 and Figures S2,S3), compound **1** was predicted to be ancistrocladinium A, a naphthylisoquinoline alkaloids produced from *Ancistrocladus tectorius* [1,2].

The peak areas of compound **1** in AT 70% ethanol extract and its BuOH fraction were 1,868,790 ± 28,096 and 11,083,858 ± 184,849, respectively. The peak area of compound **1** in the BuOH fraction was 5.9 times higher than that in the AT 70% ethanol extract.

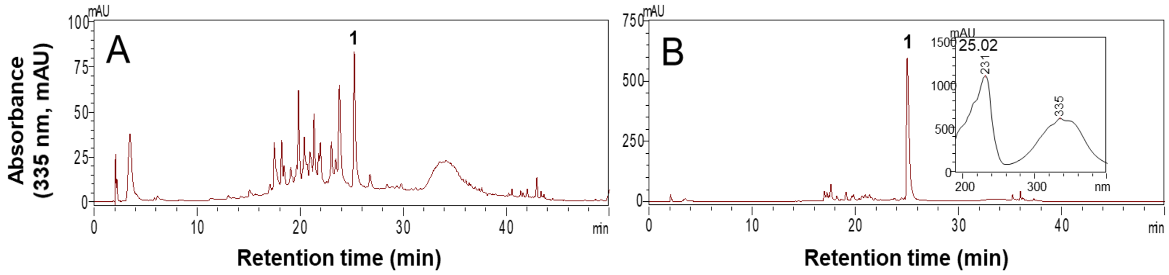

Figure S1. High-performance liquid chromatography (HPLC) profiles of the AT 70% ethanol extract and its BuOH fraction. HPLC chromatograms of (A) AT 70% ethanol extract (10 mg/mL) and (B) BuOH Fr. of AT 70% ethanol extract (10 mg/mL) were detected at 335 nm.

2. HPLC-MS/MS Analysis of AT Extract (Tables S1 and Figures S2-S4)

2.1. Materials and Method (Tabels S1)

[1] Materials

1.	Solvent	DW, ACN (B&J)
2.	Reagent	Formic acid (Aldrich)

[2] Instrument Condition

① LC Method

1.	Chromatography	Accelar UHPLC
2.	Mass spectrometry	LTQ-Orbitrap XL
3.	Column	Acquity UPLC® BEH C18, 1.7um
4.	Solvent	A: DW(0.1% FOA), B: ACN((0.1% FOA)
5.	Flow rate	400 ul/min
6.	Injection	4 μL

② MS Method

1.	Detection ion mode	Positive($[M+H]^+$)
2.	Scan range	PDA:200~600 nm; MS : *m/z* 150~1500
3.	Spray voltage	3.5 kV
4.	Capillary voltage	20V
5.	Capillary Temp.	350 ℃
6.	Software	Xcalibur

2.2. Results

[1] PDA & MS chromatogram (AT extract) (Figures S2)

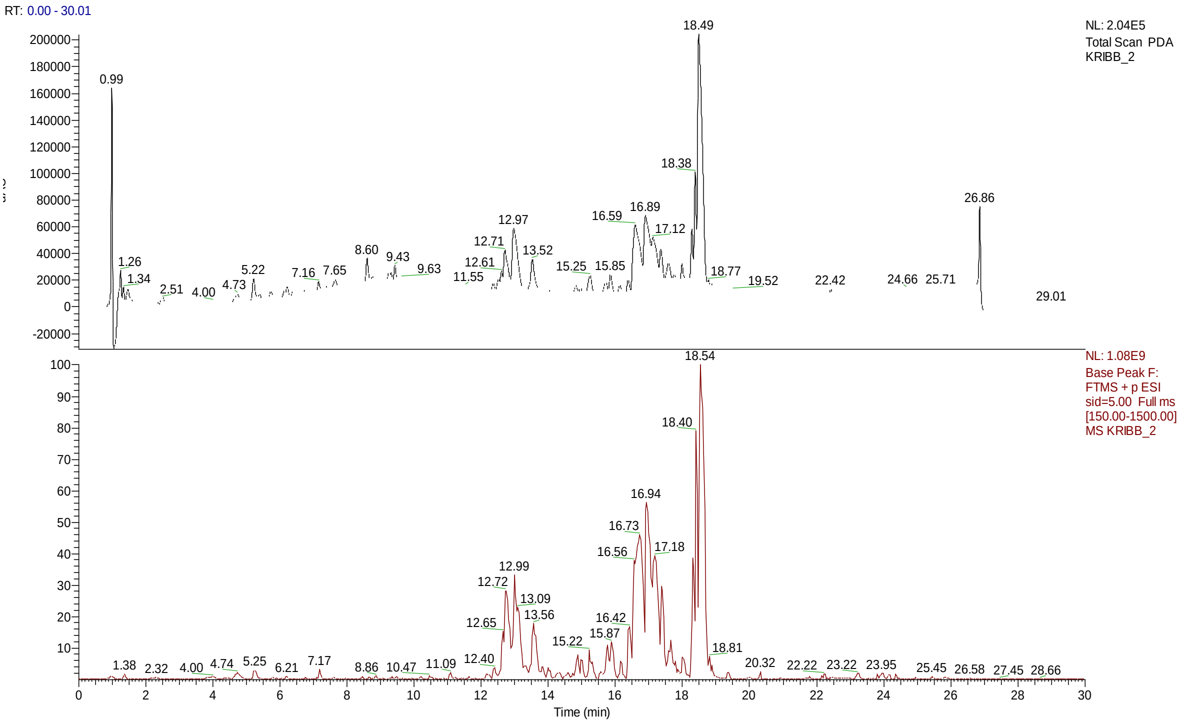

[2] PDA (AT extract, RT: 18.49) (Figures S3)

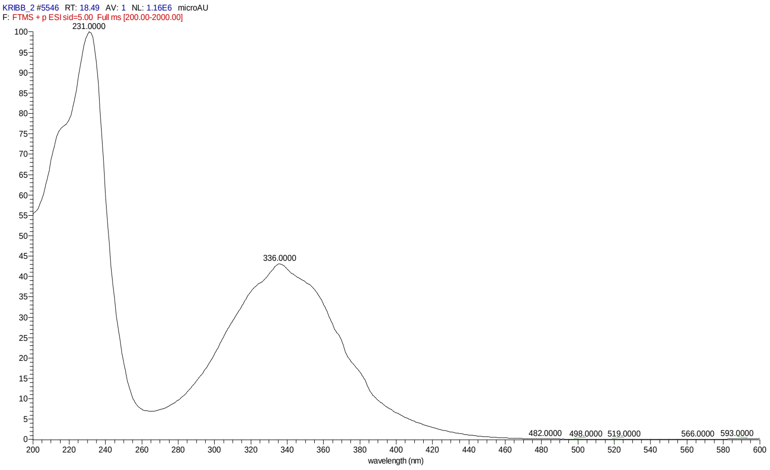

[3] MS chromatogram (AT extract, RT: 18.54) (Figures S4)

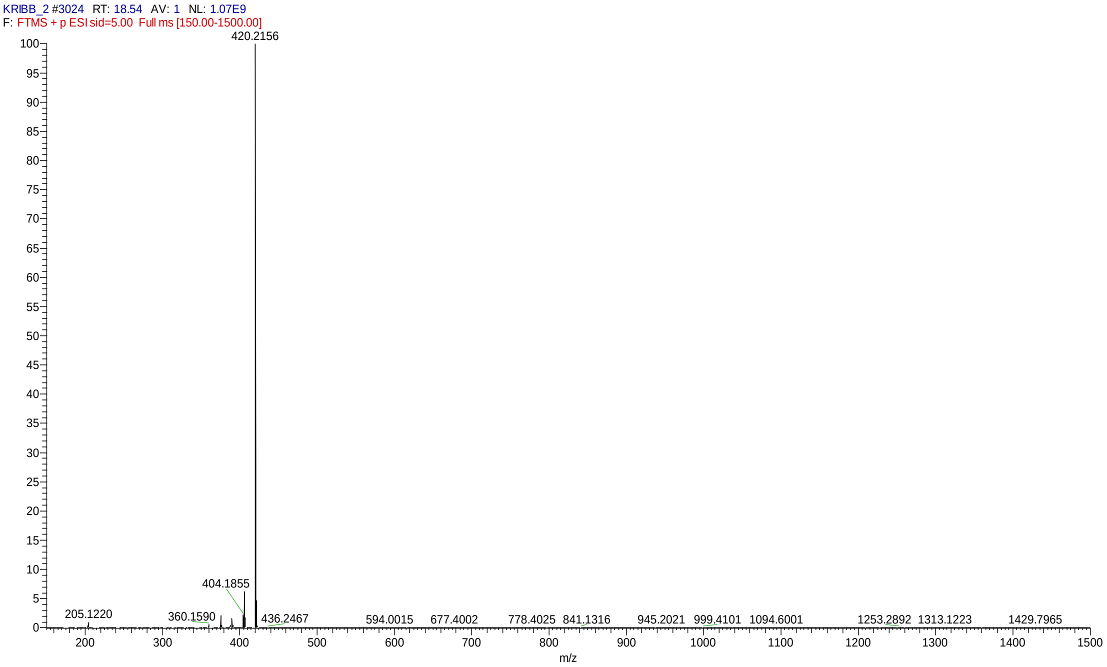

Elemental composition search on mass 420.22

m/z= 415.22-425.22

m/z	Theo. Mass	Delta (ppm)	RDB equiv.	Composition
420.2156	420.2156	0.11	13.0	$C_{24}H_{28}O_3N_4$
	420.2161	-1.09	0.5	$C_{10}H_{30}O_9N_9$
	420.2169	-3.08	12.5	$C_{26}H_{30}O_4N$
	420.2143	3.30	8.0	$C_{23}H_{32}O_7$
	420.2142	3.31	13.5	$C_{22}H_{26}O_2N_7$
	420.2174	-4.29	0.0	$C_{12}H_{32}O_{10}N_6$

[4] Reported information of expected structure **(ancistrocladinium A)**

https://pubchem.ncbi.nlm.nih.gov/compound/15984091.

PubChem About Docs Submit Contact

((3S)-2-(4,5-dimethoxy-7-methylnaphthalen-1-yl)-6,8-dimethoxy-1,3-dimethyl-3,4-dihydroisoquinolin-2-ium)

COMPOUND SUMMARY

ancistrocladinium A

PubChem CID	15984091
Structure	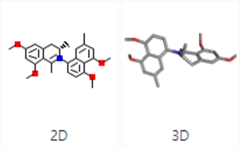
Molecular Formula	$C_{26}H_{30}NO_4^+$
Synonyms	ancistrocladinium A<br>SCHEMBL5021939<br>CHEMBL1182215<br>J3.576.241H<br>J3.576.243D<br>View More...
Molecular Weight	420.5 g/mol<br>*Computed by PubChem 2.1 (PubChem release 2021.05.07)*

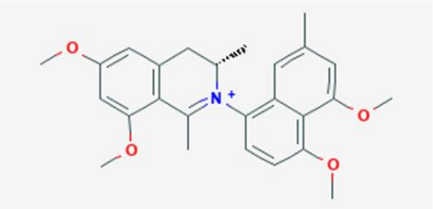

3.1 Computed Properties

Property Name	Property Value
Molecular Weight	420.5 g/mol
XLogP3-AA	5.1
Hydrogen Bond Donor Count	0
Hydrogen Bond Acceptor Count	4
Rotatable Bond Count	5
Exact Mass	420.21748344 g/mol
Monoisotopic Mass	420.21748344 g/mol

Ancistrocladinium A [(M)-5]. The isolated compound gave pale yellow crystals in a purity of >96% determined by HPLC: mp ≥230 °C (dec); $[\alpha]_D^{20}$ −6 (c = 0.05, MeOH); IR (NaCl) ν_{max} 2955, 2925, 2848, 1682, 1609, 1585, 1458, 1438, 1417, 1312, 1278, 1259, 1204, 1176, 1130, 1040, 836, 801 cm^{-1}; UV−vis (MeOH) λ_{max} 335 (log ϵ 1.65), 225 (log ϵ 2.69), 214 (log ϵ 2.73) nm; CD (MeOH) $\Delta\epsilon_{311}$ −4.4, $\Delta\epsilon_{238}$ +2.3, $\Delta\epsilon_{230}$ −3.5, $\Delta\epsilon_{214}$ +6.4; ^1H NMR (400 MHz, CD_3OD) δ 1.30 (d, $_3J$ = 7.1 Hz, 3 H, CH_3-3), 2.50 (s, 3 H, CH_3-2′), 2.52 (s, 3 H, CH_3-1), 3.13 (dd, $_2J$ = 17.4 Hz, $_3J$ = 2.5 Hz, 1 H, H_{eq}-4), 3.83 (dd, $_2J$ = 17.4 Hz, $_3J$ = 6.2 Hz, 1 H, H_{ax}-4), 3.97 (s, 3 H, OCH_3-4′), 4.01 (s, 3 H, OCH_3-5′), 4.03 (s, 3 H, OCH_3-6), 4.04 (s, 3 H, OCH_3-8), 4.25 (m, 1 H, H-3), 6.74 (s, 1 H, H-7), 6.77 (s, 1 H, H-5), 6.97 (d, $_3J$ = 8.5 Hz, 1 H, H-6′), 6.98 (s, 1 H, H-3′), 7.08 (s, 1 H, H-1′), 7.46 (d, $_3J$ = 8.5 Hz, 1 H, H-7′) ppm; ^{13}C NMR (100 MHz, CD_3OD) δ 17.2 (CH_3-3), 23.9 (CH_3-2′), 26.5 (CH_3-1), 36.7 (C-4), 58.6 (OCH_3-5′), 58.9 (OCH_3-4′), 58.8 (OCH_3-6), 58.9 (OCH_3-8), 61.3 (C-3), 100.7 (C-7), 106.6 (C-6′), 111.1 (C-5), 112.9 (C-3′), 113.8 (C-9), 114.8 (C-1′), 119.7 (C-8′), 128.9 (C-7′), 131.6 (C-10′), 133.8 (C-9′), 143.7 (C-2′), 143.9 (C-10), 161.5 (C-4′), 162.7 (C-5′), 168.1 (C-8), 172.4 (C-6), 179.6 (C-1) ppm; EIMS m/z (%) = 420.2 (12.3) $[M^+]$, 419.2 (27.9) $[M - H^+]$, 404.1 (100) $[M - CH_4^+]$; HRMS (ESI) calcd for $C_{26}H_{30}NO_4^+$ 420.21748; found 420.21750.

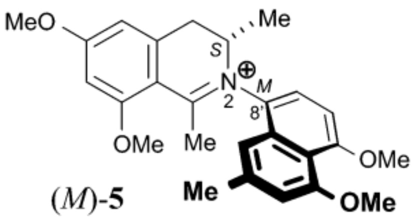

References

12. Ruangrungsi, N., Wongpanich, V.; Tantivatana, P.; Cowe, H.J.; Cox, P.J.; Funayama, S.; Cordell, G.A. Traditional medicinal plants of Thailand, V. Ancistrotectorine, a new naphthalene-isoquinoline alkaloid from *Ancistrocladus tectorius*. *J. Nat. Prod.* **1985**, *48*, 529–535.
16. Chen, Z.; Wang, B.; Qin, K.; Zhang, B.; Su, Q.; Lin, Q. Isolation and identification of alkaloids from *Ancistrocladus tectorius*. *Acta Pharm. Sin.* **1981**, *16*, 519–523.
20. Bringmann, G., Kajahn, I., Matthias Reichert, M.; E. H. Pedersen, S.E.H.; Faber, H.H.; Gulder, T.; Brun, R.; Christensen, S.B.; Ponte-Sucre,A.; Moll, H.; Heubl, G.; Virima Mudogo, M. Ancistrocladinium A and B, the First N,C-Coupled Naphthyldihydroisoquinoline Alkaloids, from a Congolese *Ancistrocladus* Species. *J. Org. Chem.* **2006**, *71*, 9348-9356.

3. Effect of AT 70% EtOH extract on body weight in HFD-fed mice

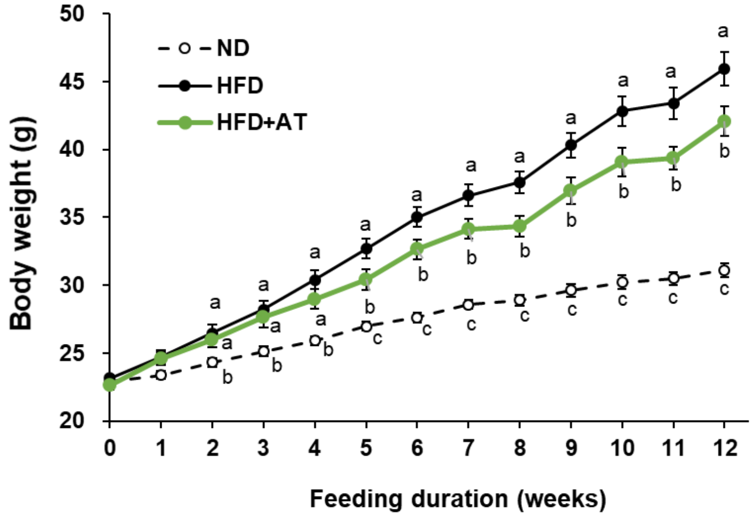

Figure S5. Effects of AT extract supplementation on change of body-weight in HFD-fed mice. Data are expressed as means ± SE. Different letters (a, b, c) within a variable are significantly different at $P < 0.05$.

4. Primer sequences used in this article are described in Table S2.

Table S2. Primers used for mRNA quantification

Gene name	Forward primer	Reverse primer
aP2	TTTCTCACCTGGAAGACAGC	TGATGCTCTTCACCTTCCTG
Adrb1	TCGGTAGATGTGCTGTGTGTGA	AGCAAACTCTGGTAGCGAAAGG
Adrb2	CCTTACCTCCTTTTTGCCTATCC	AGTCTCCTCGGTGTAACAATCGA
Cidea	TGGAAAAGGGACAGAAATGGA	TCCCGATTTCTTTGGTTGCTT
Dnm1	GAGCCAATCCATCTCAAGGTTT	TTCCCGGTAAATCCACAAGTG
Dnm2	CATCCGTGACCTTATGCCAAA	AATACAGGTAAGCCAGCAGCTCAT
Fas	TGTGAGTGGTTCAGAGGCAT	TTCTGTAGTGCCAGCAAGCT
Gapdh	ACATCATCCCTGCATCCACT	AGATCCACGACGGACACATT
Mff	CGTGGCATTTTGTCGCTTATC	AGGTCTGCGGTTTTCATCCA
Mlxipl	CAGATGCGGGACATGTTTGA	AATAAAGGTCGGATGAGGATGCT
Mfn1	TCAACACTGATGAACACGGAGAA	GAATGAAGATGTTGGGCTTGGA
Mfn2	CGAGGCTCTGGATTCACTTCA	CCAACCAGCCAGCTTTATTCC
Opa1	CCTTTGTCGCAGAGGTTTTTATTAC	AACAGGACCACGTCATTGCA
Pgc1a	GTGCAGCCAAGACTCTGTAT	GGTCGCTACACCACTTCAAT
PPARg	TGGGAGATTCTCCTGTTGAC	AGGTGGAGATGCAGGTTCTA
Prdm16	GCGCTTCGAATGTGAAAACTG	TTGGAGAACTGCGTGTAGGACTT
Srebf1	GAGCGAGCGTTGAACTGTAT	ATGCTGGAGCTGACAGAGAA
Srebf2	TCCTCCATCAACGACAAAATCA	ACTTGTGCATCTTGGCATCTGT
Ucp1	CCAGGCTTCCAGTACCATTA	GCCACACCTCCAGTCATTAA
